# Slip Spring-Based Mesoscopic Simulations of Polymer Networks: Methodology and the Corresponding Computational Code

**DOI:** 10.3390/polym10101156

**Published:** 2018-10-16

**Authors:** Grigorios Megariotis, Georgios G. Vogiatzis, Aristotelis P. Sgouros, Doros N. Theodorou

**Affiliations:** 1 School of Chemical Engineering, National Technical University of Athens (NTUA), 9 Heroon Polytechniou Street, Zografou Campus, GR-15780 Athens, Greece; gregm@mail.ntua.gr (G.M.); arissgouros@gmail.com (A.P.S.); 2 Polymer Technology, Department of Mechanical Engineering, Eindhoven University of Technology, PO BOX 513, 5600MB Eindhoven, The Netherlands; g.vogiatzis@tue.nl

**Keywords:** slip springs, crosslinked polymer networks, mesoscopic simulations, computational rheology, Brownian dynamics (BD), kinetic Monte Carlo (kMC), multiscale modeling

## Abstract

In previous work by the authors, a new methodology was developed for Brownian dynamics/kinetic Monte Carlo (BD/kMC) simulations of polymer melts. In this study, this methodology is extended for dynamical simulations of crosslinked polymer networks in a coarse-grained representation, wherein chains are modeled as sequences of beads, each bead encompassing a few Kuhn segments. In addition, the C++ code embodying these simulations, entitled Engine for Mesoscopic Simulations for Polymer Networks (EMSIPON) is described in detail. A crosslinked network of *cis*-1,4-polyisoprene is chosen as a test system. From the thermodynamic point of view, the system is fully described by a Helmholtz energy consisting of three explicit contributions: entropic springs, slip springs and non-bonded interactions. Entanglements between subchains in the network are represented by slip springs. The ends of the slip springs undergo thermally activated hops between adjacent beads along the chain backbones, which are tracked by kinetic Monte Carlo simulation. In addition, creation/destruction processes are included for the slip springs at dangling subchain ends. The Helmholtz energy of non-bonded interactions is derived from the Sanchez–Lacombe equation of state. The isothermal compressibility of the polymer network is predicted from equilibrium density fluctuations in very good agreement with the underlying equation of state and with experiment. Moreover, the methodology and the corresponding C++ code are applied to simulate elongational deformations of polymer rubbers. The shear stress relaxation modulus is predicted from equilibrium simulations of several microseconds of physical time in the undeformed state, as well as from stress-strain curves of the crosslinked polymer networks under deformation.

## 1. Introduction

Multiscale modeling is a current trend in the field of molecular simulations, since combining information from different levels of description allows predicting material properties which depend on broad ranges of length and time scales at reasonable computational cost. In the case of polymer systems, their long relaxation times pose severe obstacles for molecular dynamics (MD) simulations at the atomistic level. This may be alleviated by coarse-graining, which entails a reduction in the number of degrees of freedom used to describe a given macromolecule. Coarser representations of polymer chains involving fewer degrees of freedom are invoked by mesoscopic (or coarse-grained) simulation methods, capable of accessing length scales in the 10 nm–1 μm range and time scales in the μs to ms range [1,2]. A well-known coarse-grained model was developed by Kremer and Grest in the early 1990s [3]. Systematic coarse-graining methods, such as Iterative Boltzmann Inversion (IBI), have been utilized for the development of coarse-grained models of polymers [4,5,6,7,8,9]; in the case of IBI, a set of target structural and thermodynamic properties of an underlying atomistic model is reproduced at the new, less-detailed, level of description. Another promising approach is the integral equation coarse-graining method that employs the first-principles Ornstein–Zernike equation [10]. Furthermore, an energy renormalization scheme for achieving temperature transferable coarse-grained models of polymer materials was recently published [11]. One of the main characteristics of polymer melts, concentrated polymer solutions and crosslinked polymer networks is the entanglement effect, occurring as a result of the *uncrossability* of polymer chains, which causes complicated topological constraints [12,13,14,15,16]. Short linear polymeric chains in the melt state are not entangled and their long-time dynamics is adequately described by the well-known Rouse model which envisions a chain as a string of Brownian particles connected by harmonic springs moving in a viscous medium and neglects excluded volume effects and hydrodynamic interactions [17]. As the molecular weight of chains in a melt increases, a crossover point (*M*_c_) from the unentangled to the entangled regime is observed. The Zimm model, published a few years after the Rouse model, takes into account hydrodynamic interactions and is appropriate for dilute polymer solutions [18]. The tube model, which considers a single long chain in a mean field formed by the remaining chains, envisions that the chain is confined by entanglements within a tube-like region which restricts its lateral motion [19,20,21,22]. The contour of the tube is known as the primitive path [12]. This model becomes more realistic by introducing additional relaxation mechanisms such as contour length fluctuation and constraint release [23,24,25]. The entanglement effect is also modeled by slip link models, in which a number of discrete points (entanglements) confine the lateral motion of the polymer chain, while the chain is able to slide through them [26,27,28,29,30,31]. An interesting variation of the slip link model is the so-called (single-chain) slip spring model, introduced by Likhtman in 2005 [32]; the lateral motion of the chain is restricted by links connected by springs to links on other chains, with slip springs being created or destroyed at chain ends. The concepts of slip springs and slip links have been widely employed in multi-chain mesoscopic simulations for the calculation of viscoelastic properties of polymers [33,34,35,36,37,38,39,40,41,42,43,44,45,46,47,48,49,50,51]. In most of these studies, the movement of the coarse-grained entities in space is tracked by Brownian dynamics (BD), while some recent studies apply dissipative particle dynamics (DPD) [43,44,47], which is also widely utilized for simulations at the mesoscale. An additional inherent characteristic of such multi-chain simulations is the hopping, creation and destruction processes inevitably needed for the slip links or slip springs. Such processes may be described by Monte Carlo or one-dimensional Brownian dynamics schemes.

Polymer chains may be interconnected into a three-dimensional network with the connection points being referred to as permanent links (or crosslinks). These may be formed by chemical bonds (e.g., sulfur bridges) or physical aggregates. If the crosslinked polymer chains are highly mobile and flexible, the polymer network manifests very high deformability and practically complete recovery [52]. Such polymeric materials are known as rubber-like materials or elastomers, while their elastic behavior is called rubber-like elasticity. The term *rubber-like* is used to indicate the similarity of these materials to natural rubber, which consists mainly of *cis*-1,4-polyisoprene (*cis*-PI) [52,53]. As far as the creation of crosslinked polymer networks is concerned, this is often realized through random crosslinking or end-linking. A number of molecular theories have been introduced for the interpretation of rubber-like elasticity, such as the affine network model, the phantom network model, the constraint junction model, the diffuse constrained model, the slip link model, the tube model of Edwards, and the nonaffine tube model [19,22,27,54,55,56,57,58,59,60,61,62,63,64,65,66,67]. The ideas of slip link and confining tube were combined in the *slip-tube* model developed by Rubinstein and Panyukov [68]. A concise discussion of rubber-like elasticity can be found in [52]. The concepts of slip links and slip springs have been successfully introduced in multi-chain mesoscopic simulations of crosslinked polymer networks [35,36,48], which differ from the corresponding multi-chain simulations of polymer melts in the existence of permanent links that do not allow the connecting neighboring chains to slide through them. At more detailed levels of description, crosslinked polymer networks have also been modeled through atomistic simulations; time and length scales are rather limited in such approaches [15,69,70,71,72,73], however. Another approach to molecular simulation of elastomeric materials is coarse-grained molecular dynamics, in which the network is made up of coarser entities than atoms and consequently longer time and length scales are covered in comparison with those of the atomistic models [74,75]. An interesting hierarchical multiscale methodology concerning epoxy polymer networks has been published by Gavrilov et al. [76].

The aim of the present study is twofold: first, to develop a mixed particle and field-based Brownian dynamics/kinetic Monte Carlo simulation approach to crosslinked polymer networks in the absence or presence of externally imposed mechanical deformation, and secondly to describe the Engine for Mesoscopic Simulations for Polymer Networks (EMSIPON) code that has been developed to implement this approach in mesoscopic simulations of polymer melts and elastomeric materials under equilibrium and non-equilibrium conditions. While, in principle, our methodology could be applied for simulating glassy polymers, it would be extremely inefficient because of the very broad spectra of time scales that would have to be covered and would require special provisions to capture the hierarchically constrained dynamics of a glass. The representation has been designed so as to preserve chemical specificity; yet being able to access length scales on the order of 10–100 nm and time scales on the order of 1–20 ms. Chains are represented as sequences of beads, each bead consisting of 5–12 Kuhn segments. A new mesoscopic free energy function is introduced which incorporates bonded, as well as non-bonded interactions and yields realistic predictions of the thermodynamic properties (e.g., isothermal compressibility) of the simulated systems. Chemical crosslinks are represented explicitly, while the entanglement effect is introduced by slip springs whose hopping, creation and destruction are tracked by a kinetic Monte Carlo (kMC) scheme obeying the condition of microscopic reversibility. A principal aim is to compute viscoelastic properties. *cis*-PI crosslinked networks serve as a test system. In addition, we pay attention to the description of the algorithms being used for the crosslinking of monodisperse polymer melts of linear chains.

The already fully developed and tested method (in polymer melts under equilibrium [49] and flow conditions [50]) can track the time evolution of polymer melts at long timescales (in the order of the longest relaxation time). Using as starting point a well equilibrated coarse-grained configuration of the system, the simulation setup is versatile both in terms of systems that can be studied and problems that can be addressed. All necessary interaction parameters are deduced by a rigorous and systematic bottom-up approach, starting from atomistic simulations which alone fall short in tackling the long time-scales of polymer melts and rubbers. All ingredients of our model are based on either more detailed (atomistic) simulations or experimental findings. For every parameter introduced, we have thoroughly explained its physical meaning and provided rigorous guidelines in reference [49].

The paper is organized as follows: First, all models and methods employed for the mesoscopic simulations of the polymer networks are detailed in Section 2, while a description of the EMSIPON code and its advantages over the currently extensive literature, which carries out the presented methodology, is given in Section 3. Next, simulation details are provided in Section 4. Section 5 presents results from the BD/kMC simulations of *cis*-PI networks in the deformed and undeformed states. Section 6 summarizes and discusses the most important aspects of the work.

## 2. Model and Methods

### 2.1. Model and Free Energy Function

**Figure 1:**
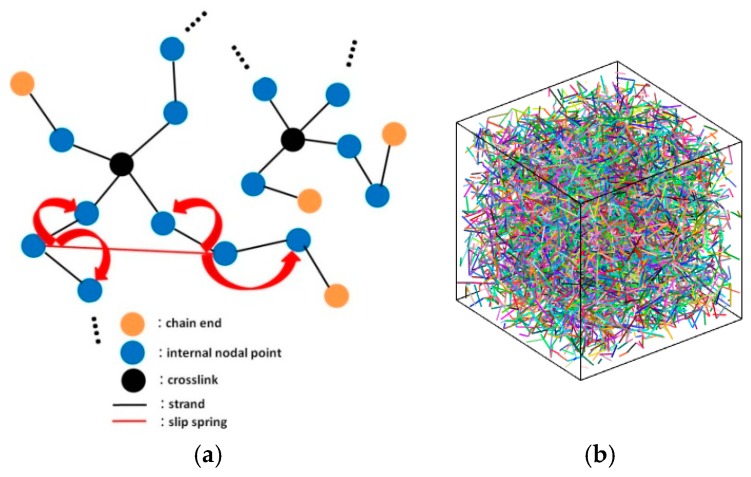
(**a**) Representation of a polymer network at the coarse-grained level. End beads, internal beads, and crosslink beads are shown as orange, blue, and black circles, respectively. A slip spring is shown as a red line. Red arrows indicate possible jumps that can be executed by the ends of the slip spring. (**b**) Visualization of the crosslinked *cis*-PI with all nodal points being folded back into the simulation box. The different colors of strands are chosen randomly.

Initial configurations for the polymer as a melt of linear chains are obtained by a field-theory inspired Monte Carlo (FTi-MC) simulation of a coarse-grained model, wherein chains are represented as freely jointed sequences of Kuhn segments subject to a coarse-grained Helfand Hamiltonian that prevents the density from deviating from its mean value [77,78]. Coarse-graining from the freely jointed chain to a bead-spring model is applied before crosslinking and involves lumping sets of successive Kuhn segments along a chain into single beads at regular intervals along the polymer chain. Strands, which are chain sections between two successive beads, are modeled as springs representing the entropic elasticity of the polymer. More details about the creation of initial configurations of polymer melts at the coarser level of description can be found elsewhere [49]. At this new level of description, the polymer is envisioned as a network of strands connecting crosslink points, end points (or chain ends), and internal nodal points (see also Figure 1a). The entanglement effect is introduced through slip springs with each slip spring connecting either two spatially neighboring beads on two different chains or two beads belonging to the same chain. A chain may slide past a slip spring through which it passes. The number of entanglements (slip springs) is usually chosen to be consistent with the molar mass between entanglements, *M*_e_, in the linear melt. The crosslinks are introduced according to a prescribed density and mode of crosslinking, as described in Section 2.2, leading to the formation of a crosslinked polymer network. A graphical representation of such a system is provided in Figure 1b.

In the context of this study, the Helmholtz energy (potential of mean force, effective interaction energy) of the mesoscopic model system is written as a function of the coarse-grained degrees of freedom in the form of a sum of three contributions:

(1) $$A=A_{\mathrm{entropy}\;\mathrm{springs}}+A_{\mathrm{slip}\;\mathrm{springs}}+A_{\mathrm{nb}}\;$$

The first term on the right-hand side of Equation (1) is the contribution from the conformational entropy of strands. It is computed from the Gaussian expression:

(2) $$A_{\mathrm{entropy}\;\mathrm{springs}}^{}=\sum_{ij}\frac{3}{2}k_{B}T\;\frac{r_{ij}^{2}}{n_{ij}b^{2}}\;$$

where

*b*: Kuhn length, indicative of the conformational stiffness of the polymer and dependent on the chain chemical constitution. In the case of *cis*-PI, *b* is equal to 9.58 Å [79].
*k*_B_: Boltzmann constant;
$r_{ij}$: Length of end-to-end vector of the strand;
$n_{ij}$: Number of Kuhn segments in the strand;
*ij*: Strand connecting beads *i* and *j*.

Alternatively, one can resort to non-linear entropic springs that are useful for very large deformations of crosslinked polymer networks or for non-equilibrium mesoscopic simulations of polymer melts under strong flows [50]. The alternative expressions that are available in the EMSIPON code are detailed in references [50,80]. In brief, the force exerted between two beads connected via a non-linear spring is computed by the following equations

(3) $$F_{ij}=-\frac{k_{B}T}{b}L^{-1}(y_{ij}){\overset{}{\hat{r}}}_{ij}=-\frac{k_{B}T}{b}L^{-1}(y_{ij})\frac{r_{i}-r_{j}}{r_{ij}},\;y_{ij}=\frac{r_{ij}}{L_{ij}}=\frac{r_{ij}}{n_{ij}b}\;$$

with ${\hat{r}}_{ij}$ being a unit vector along strand *ij* pointing from *j* to *i* and *L_ij_* being the maximum length of the strand. $L^{-1}(y_{ij})$ and $y_{ij}$ denote the inverse Langevin function and the relative stretch of the strand connecting nodal points *i* and *j*, respectively. In this case, the Helmholtz energy of the entropic springs of a polymer network is provided by Equation (4):

(4) $$A_{\mathrm{entropy}\;\mathrm{springs}}^{}=\sum_{ij}n_{ij}k_{B}T\int_{0}^{r_{ij}/L_{ij}}L^{-1}(y_{ij})dy_{ij}\;$$

In the context of this study and reference [50], the inverse Langevin function is not approximated by the commonly employed approximants (e.g., Warner’s approximant), but by the expressions proposed by M. Kröger [80]. The Helmholtz energy of slip springs, which are included to account for the entanglement effect, is here described by the simple Gaussian equation:

(5) $$A_{\mathrm{slip}\;\mathrm{springs}}=\sum_{kl}A_{\mathrm{pair}}^{\mathrm{sl}}=\sum_{kl}\frac{3k_{B}T}{2b_{\mathrm{sl}}^{2}}{\left(r_{k}-r_{l}\right)}^{2}\;$$

Equation (5) involves a sum over all slip springs *kl* whose ends have position vectors $r_{k}$ and $r_{l}$. In the context of our approach, none of the two ends of a slip spring is allowed to attach to a crosslink. There is an additional parameter $b_{\mathrm{sl}}$ controlling the stiffness of the slip spring which can be set equal to the entanglement tube diameter, *α*_pp_, of the simulated polymer [49,81]. The free energy term $A_{\mathrm{slip}\;\mathrm{springs}}$ computed by Equation (5) refers to linear (Gaussian) springs; non-linear ones may be utilized instead, in the same fashion as $A_{\mathrm{entropy}\;\mathrm{springs}}^{}$ may be calculated either by Equation (4) or Equation (2). Non-linear slip-springs must be used when strong flows and large deformations are of interest [50].

To deal with non-bonded interactions in the polymer network representation, a non-bonded Helmholtz energy contribution is included:

(6) $$A_{\mathrm{nb}}=\int d^{3}r\;a_{\mathrm{vol}}^{\mathrm{ex}}[\rho (r)]\;$$

In Equation (6), ρ(r) is the local density at position **r** and $a_{\mathrm{vol}}^{\mathrm{ex}}(\rho )$ is an excess free energy density (Helmholtz energy per unit volume) relative to an ideal gas of chains, which is obtained from the local density using an equation of state (EOS). Here we make use of the Sanchez–Lacombe EOS [82]:

(7) $$\tilde{P}+{\tilde{\rho }}^{2}+\tilde{T}\left[\ln(1-\tilde{\rho })+\left(1-\frac{1}{r}\right)\tilde{\rho }\right]=0\;$$

(8) $$\tilde{T}=\frac{T}{T^{*}},\tilde{\rho }=\frac{\rho }{\rho^{*}},P=\frac{P}{P^{*}},r=\frac{M}{\rho^{*}}\frac{P^{*}}{RT^{*}}\;$$

where the temperature, pressure and mass density of the system are symbolized by *T*, *P* and *ρ*, respectively and *M* is the molar mass of a chain. T*, P*, and ρ* are parameters dictated by the energy of non-bonded attractive interactions between polymer segments, the hard-core volume of a segment, and the mass of a segment in the lattice fluid picture underlying the EOS. The excess pressure relative to an ideal gas of chains at the same density and temperature satisfies:

(9) $${\tilde{P}}^{\mathrm{ex}}+{\tilde{\rho }}^{2}+\tilde{T}\left[\ln(1-\tilde{\rho })+\tilde{\rho }\right]=0\;$$

The corresponding excess free energy density is given by the following equation:

(10) $$\frac{1}{P^{*}}a_{\mathrm{vol}}^{\mathrm{ex}}(\rho )=\tilde{T}\tilde{\rho }-{\tilde{\rho }}^{2}+\tilde{T}\left(1-\tilde{\rho }\right)\ln\left(1-\tilde{\rho }\right)\;$$

Local density is resolved only at the level of entire cells, defined by passing an orthogonal grid through the entire system. Equation (6) is approximated by:

(11) $$A_{\mathrm{nb}}=\sum_{\mathrm{cells}}V_{\mathrm{cell}}^{}\;a_{\mathrm{vol}}^{\mathrm{ex}}(\rho_{\mathrm{cell}})\;$$

where $V_{\mathrm{cell}}^{}$ = $L_{x}^{\mathrm{grid}}L_{y}^{\mathrm{grid}}L_{z}^{\mathrm{grid}}$ is the volume of the rectangular parallelepiped cell. The cell density $\rho_{\mathrm{cell}}$ is defined based on the nodal points in and around the cell and in the following we will assume that,

(12) $$R_{j}<\min(L_{x}^{\mathrm{grid}},L_{y}^{\mathrm{grid}},L_{z}^{\mathrm{grid}})\;$$

where $R_{j}$ is the characteristic size of the nodal points. The aforementioned inequality means that the adopted scheme is restricted to consider only the first neighbors of a grid cell. The full description of the free energy contribution due to non-bonded interactions is given in a previous study by Vogiatzis et al. [49].

It is also remarked that the total Helmholtz energy, *A*, may be modified accordingly to describe more complex polymer systems by introducing additional terms in Equation (1). For instance, Sgouros et al. recently included an additional free energy term corresponding to the bending angles between three successive beads along the polymer chains [50]. Furthermore, the authors of the present article are now extending the methodology described herein, along with the EMSIPON code, to simulate polymer melts in the presence of planar surfaces (e.g., graphene plates), solid inclusions (e.g., silica nanoparticles) or vacuum (e.g., formation of droplets, free films, bubbles, etc.) at the mesoscale. In the aforementioned cases, extra terms are included in the total Helmholtz energy function for the description of solid/polymer, solid/solid and polymer/vacuum interactions.

### 2.2. Crosslinking

In the context of this study, initial configurations of crosslinked *cis*-PI networks are created either by random crosslinking or by end grafting of linear melt configurations.

**Figure 2:**
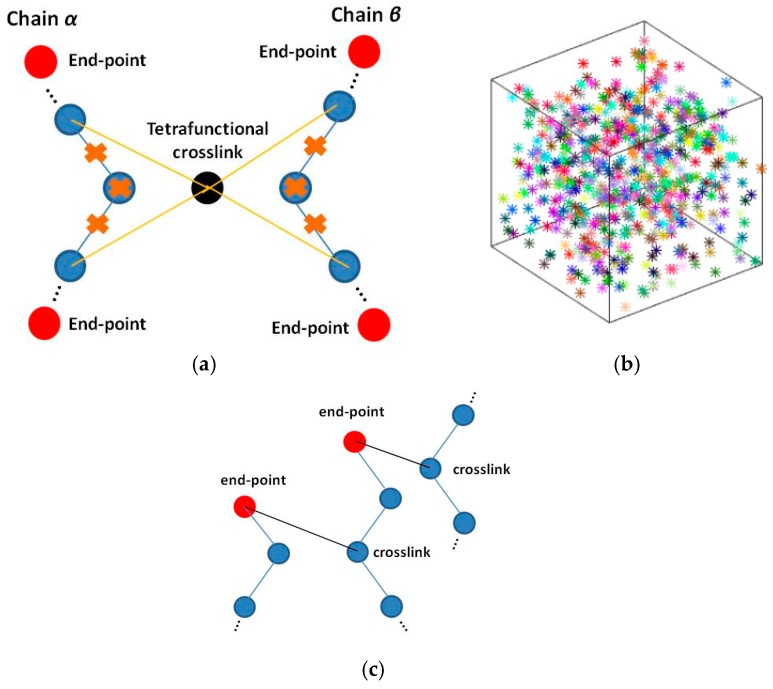
(**a**) Schematic representation of a tetrafunctional crosslink created by random crosslinking; (**b**) visualization of 600 crosslinks randomly dispersed in a cubic simulation box of edge length 30 nm (after the implementation of random crosslinking); (**c**) crosslinking through end-grafting: conversion of an internal nodal point to a trifunctional crosslink which is connected with an end-point of another polymer chain.

In random crosslinking, a prescribed number of points (*n*_c_) is generated at random in the simulation box to serve as the permanent links of the network. A crosslink connects two different polymer chains with four strands emanating from it (functionality *φ* = 4). In our simulation, at each generated crosslink point a pair of beads belonging to two neighboring linear chains is captured. Note that all end-points are excluded from this kind of crosslinking construction. The construction around a randomly generated crosslink point proceeds as follows. First, the two beads to be captured are identified. To this end, we search for beads lying inside a sphere of prescribed radius *R*_c_, which is a parameter of the EMSIPON code, around the randomly selected crosslink. Next, the beads lying within the sphere are sorted according to their distance from the crosslink point. The closest bead is selected and all other beads belonging to its chain are excluded from selection; this means that all crosslinks are intermolecular. Of the remaining beads, the one lying closest to the crosslink point is selected. The two beads identified in this way are merged into a single crosslink bead centered at the position of the crosslink point. The four strands originally emanating from the two beads are brought together at the newly created crosslink bead. A schematic representation of this procedure is provided in Figure 2a in which the symbol (X) indicates the strands and nodal points of the original linear chains that are deleted. It is also seen that the first neighbors of the deleted beads participate in the local reconstruction of connectivity. The crosslink bead is shown as a black circle and the new strands as orange lines. Overall, crosslinking implemented in this way leaves the number of strands in the system constant, while reducing the total number of nodal points by *n*_c_. A visualization of a simulation box containing 600 randomly dispersed crosslink points is seen in Figure 2b. Before proceeding to the detailed description of the other kind of crosslinking, it should be mentioned that random crosslinking is of great importance from an industrial point of view and may be achieved by high energy irradiation, copolymerization (requirement for comonomers of functionality greater than two), and sulfur and peroxide cures [52].

As regards the end grafting, it is employed for the creation of a trifunctional crosslinked polymer network without dangling ends, starting from an equilibrated monodisperse or polydisperse polymer melt of linear chains. It is also pointed out that the implementation of this kind of crosslinking to branched or star polymer melts is straightforward. In more detail, each end-point of an original linear polymer chain is connected to one of the closest internal nodal points of another chain via the addition of a new strand. Thus, trifunctional crosslinks are formed (functionality *φ* = 3). By construction of this algorithm, the total number of crosslinks equals $n_{c}=2n_{\mathrm{chains}}=n_{\mathrm{end}\text{-}\mathrm{points}}$. The average number of subchains between crosslinks per initial linear chain is $\varphi n_{c}/\left(2n_{\mathrm{chains}}\right)=3$. For each end-point, we search for internal nodal points of other polymer chains inside a sphere of prescribed radius *R*_c_. When a nodal point is turned into a crosslink, it is no longer available for being connected with other end-points during the end grafting procedure. The same holds for the initial end-points that are connected with trifunctional crosslinks. Upon completion of this kind of crosslinking, the number of nodal points remains constant while the number of strands increases by $n_{\mathrm{end}\text{-}\mathrm{points}}$. The main advantage of this crosslinking is that no dangling chain ends remain. A graphical representation of end-grafting is given in Figure 2c, in which the end-point is depicted in red color and the new strand is sketched by a black line connecting the aforementioned end-point with the internal nodal point (blue color) of a neighboring chain. The entropic springs of the initial linear chains are in blue color as well.

### 2.3. Brownian Dynamics and Kinetic Monte Carlo (BD/kMC) Scheme

The temporal evolution of our polymer network configurations is tracked by Brownian dynamics (also known as position Langevin dynamics), which is Langevin dynamics in the high friction limit. The general form of the equation of motion for each nodal point *i* is given by the following stochastic differential equation of first order:

(13) $$\frac{dr_{i}\left(t\right)}{dt}=\frac{1}{m_{i}\xi_{i}}f_{i}+u_{i}(t),\;f_{i}=-\nabla_{r_{i}}A\;$$

$r_{i}$ is the position vector of nodal point *i*, $u_{i}(t)$ is the random velocity imparted to it and $f_{i}$ is the total systematic force exerted on the nodal point in question. In addition, this force is connected with the Helmholtz free energy function *A*, as defined by Equation (1), via the second relation of the above set of equations. The product $\zeta_{i}=m_{i}\xi_{i}$ is a friction factor for node *i*, with $\xi_{i}$ having the dimensions of inverse time. In the context of this study, all nodal points are assumed to be of equal size and therefore experience the same friction coefficient, $\zeta_{0}$. According to the fluctuation-dissipation theorem, the components of the random velocities $u_{ik}(t)$ with *k* ∈ {1,2,3}, are delta-correlated, with variance:

(14) $$\langle u_{ik}(t)u_{jl}(t+\tau )\rangle =\frac{2k_{B}Τ}{m_{i}\xi_{i}}\delta_{ij}\delta_{kl}\delta (\tau )\;$$

Here *δ_ij_* and *δ*(*τ*) are the Kronecker’s delta and the Dirac delta function, respectively. The set of position Langevin equations is solved numerically, as shown in the next equation which is a first order Euler scheme, marching along the time axis in steps of magnitude $\Delta t>1/\xi_{i}$:

(15) $$r_{i}(t+\Delta t)=r_{i}(t)+\frac{\Delta t}{m_{i}\xi_{i}}f_{i}(t)+\eta_{i}(t)\;$$

An alternative numerical scheme is given by the following equation in which the first derivative of the force is included [83]:

(16) $$r_{i}(t+\Delta t)=r_{i}(t)+\frac{\Delta t}{m_{i}\xi_{i}}f_{i}(r)+\frac{1}{2}\frac{\Delta t^{2}}{m_{i}\xi_{i}}{\dot{f}}_{i}(r)+\eta_{i}(t)\;$$

Both numerical schemes are available in the EMSIPON code. In the last two equations, $\eta_{i}(t)$ are random displacement vectors with their components obeying a Gaussian distribution with time correlation functions conforming to the following equation:

(17) $$\langle \eta_{ik}(t)\eta_{jl}(t+n\Delta t)\rangle =\frac{2k_{B}T}{m_{i}\xi_{i}}\Delta t\delta_{ij}\delta_{kl}\delta_{0n}\;$$

A detailed analysis of the numerical methods employed for Brownian dynamics can be found in reference [84].

**Figure 3:**
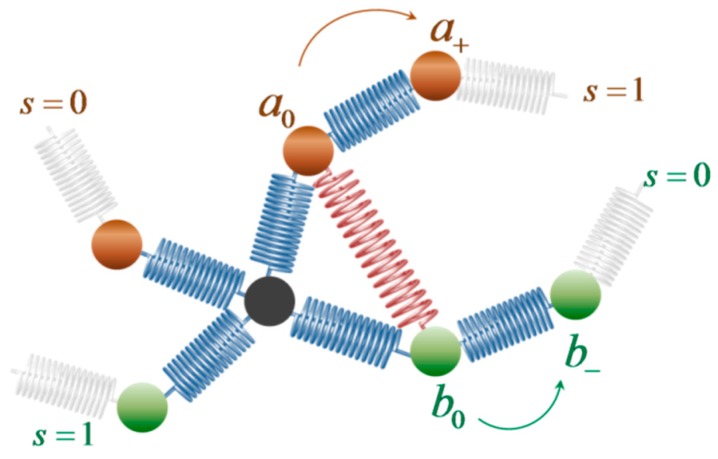
Schematic representation of the hopping scheme for slip springs along the chain contour in the event of crosslink presence in the polymer network (c.f. Figure 2 in reference [49] for the case of a polymer melt). All possible jumps are between first-neighbor beads only, whereas double jumps are not allowed.

As far as the kMC scheme is concerned, it was introduced in a previous work of the authors concerning mesoscopic simulations of *cis*-PI polymer melts [49]. In summary, slip springs are designed to mimic the entanglement effect in polymer melts and crosslinked polymer networks and their hopping, creation and destruction processes are governed by a discretized kMC scheme that fulfills the detailed balance condition (or microscopic reversibility). A slip spring may connect two internal nodal points of two different polymer chains or two nodal points belonging to the same polymer chain and can be stochastically destroyed when one of the nodes it connects is an end-point. As far as the creation process is concerned, which is initiated at the chain ends as well, two schemes have been developed: a constant number of slip springs scheme in which when one slip spring is destroyed another one is created, and a fluctuating number of slip springs scheme. In the latter, the chemical potential of slip springs is constant and hence a grand canonical ensemble is sampled with respect to the number of slip springs. Chain slippage corresponds to the hopping of a slip spring to an adjacent bead along the contour of the chains it connects, without any displacement of the beads. In elastomeric materials, the hopping scheme does not allow a slip spring to attach to a crosslink. Thus, when one or more first neighbors of a slip spring end are crosslinks, the possible discrete jumps of the considered slip spring are reduced. In the extreme case where all first neighbors of a slip spring are permanent links, the ends of this slip spring do not change as simulation time elapses. The hopping scheme in the presence of crosslinks in the polymer network is presented schematically in Figure 3. The ends of the slip spring connect beads $a_{0}$ and $b_{0}$ along chains a and b. In the context of our scheme, an individual jump of one end of a slip spring along the chain backbone, e.g., from bead $a_{0}$ to bead $a_{+}$, takes place with rate:

(18) $$k_{\mathrm{hopping}}=\nu_{\mathrm{diff}}\exp\left(\frac{A_{\mathrm{pair}}^{\mathrm{sl}}\left(r_{\alpha_{0}b_{0}},T\right)}{k_{B}T}\right)\;$$

$\nu_{\mathrm{diff}}$ is the only adjustable parameter of the model described in this section. The probabilities of the elementary events for the hopping scheme of slip springs, either in the presence or absence of crosslinks, are given by the product $p_{\mathrm{hop}}=k_{\mathrm{hopping}}\Delta t_{\mathrm{KMC}}=k_{\mathrm{hopping}}n_{\mathrm{KMC}}\Delta t$, where $\Delta t_{\mathrm{KMC}}$ and $n_{\mathrm{KMC}}$ are the time step of the hopping scheme and the number of Brownian dynamics steps between two successive kMC steps, respectively.

### 2.4. Stress Tensor and Deformations

In the case of a polymer melt or a crosslinked polymer network, with the coarse-grained Helmholtz free energy of Equation (1), the stress tensor $\tau_{\alpha \beta }${α, β = *x*, *y*, *z*} has two contributions, obtained from the springs and non-bonded interactions, and is written as a sum of two terms:

(19) $$\tau =\tau_{\mathrm{springs}}+\tau_{\mathrm{nb}}\;\mathrm{or}\;\tau_{\alpha \beta }=\tau_{\alpha \beta ,\mathrm{springs}}+\tau_{\alpha \beta ,\mathrm{nb}}^{}\;$$

As regards the computation of the two components on the right-hand side of the previous equations, we make use of the following general expression for the stress tensor [85,86,87]:

(20) $$\tau =\rho F\cdot {\left(\frac{\partial \left(A/m\right)}{\partial F}\right)}^{T}=\rho F\cdot {\left(\frac{\partial \left(A_{\mathrm{springs}}/m\right)}{\partial F}\right)}^{T}+\rho F\cdot {\left(\frac{\partial \left(A_{\mathrm{nb}}/m\right)}{\partial F}\right)}^{T}\;$$

The tensorial quantity F is the deformation gradient tensor, which is widely employed in continuum mechanics. We assume that all nodal points are transformed homogeneously according to **F**; evidence that such an assumption is realistic at the level of the nodal points invoked here comes from atomistic simulations [71]. Then, by applying Equation (20), the stress tensor components for the contribution from springs (both entropic and slip springs) and non-bonded interactions are given by the next two equations [49]:

(21) $$\begin{array}{c} \tau_{\alpha \beta ,\mathrm{springs}}^{}=\frac{3k_{B}T}{VNb^{2}}\sum_{i=1}^{N_{\mathrm{chains}}}\sum_{j=1}^{N_{\mathrm{beads}}-1}\left(r_{i\alpha }\left(j+1\right)-r_{i\alpha }\left(j\right)\right)\left(r_{i\beta }\left(j+1\right)-r_{i\beta }\left(j\right)\right)+\\ \frac{3k_{B}T}{Vb_{\mathrm{sl}}^{2}}\sum_{i=1}^{N_{\mathrm{slip}\text{-}\mathrm{springs}}}\left(\left(r_{i\alpha }\left(2\right)-r_{i\alpha }\left(1\right)\right)\left(r_{i\beta }\left(2\right)-r_{i\beta }\left(1\right)\right)\right) \end{array}$$

(22) $$\tau_{\alpha \beta ,\mathrm{nb}}^{}\left(\left\{\rho_{\mathrm{cell}}\right\},T\right)=\left\{ \begin{array}{l} -\frac{1}{V}\sum_{k\;\in \;\mathrm{cells}}P^{\mathrm{ex}}\left(\rho_{\mathrm{cell},k},T\right)\;V_{\mathrm{cell},k}^{},\;\alpha =\beta \\ 0,\;\alpha \neq \beta \end{array}\right.\;$$

In the double sum of Equation (21) $r_{i\alpha }\left(j\right)$ stands for the *α* component of the position vector of bead *j* of chain *i*. In the single sum of Equation (21) $r_{i\alpha }\left(1\right)$ and $r_{i\alpha }\left(2\right)$ stand for the *α* components of the position vectors of the two ends of slip spring *i*. The computation of the excess pressure $P^{\mathrm{ex}}\left(\rho_{\mathrm{cell},k},T\right),$ relative to an ideal gas of chains at the same temperature and density, in the sum of Equation (22) is accomplished by invoking the Sanchez–Lacombe EOS, Equation (9). It has to be noted that we resort to Equation (20) because of the field theoretic nature of non-bonded interactions in our model; Equation (21) can also be derived from the virial theorem. An interesting consequence of Equation (22) is that the off-diagonal elements of $\tau_{\mathrm{nb}}$ are equal to zero and, therefore, there is no contribution from non-bonded interactions to shear stress and viscosity in a polymer melt.

One of the main reasons for employing the stress tensor is the computation of stress relaxation function (or stress relaxation modulus) G(t) defined by the equation:

(23) $$G\left(t\right)=\frac{V}{3k_{B}T}\left[\langle \tau_{xy}\left(t_{0}+t\right)\tau_{xy}\left(t_{0}\right)\rangle +\langle \tau_{yz}\left(t_{0}+t\right)\tau_{yz}\left(t_{0}\right)\rangle \langle \tau_{zx}\left(t_{0}+t\right)\tau_{zx}\left(t_{0}\right)\rangle \right]\;$$

There is another expression in the literature computing the stress relaxation function, which involves the normal stress differences $N_{xy}=\tau_{xx}-\tau_{yy}$, $N_{xz}=\tau_{xx}-\tau_{zz}$, $N_{yz}=\tau_{yy}-\tau_{zz}$ as well [88,89]. All results presented herein are based on Equation (23) which is more straightforward, with a firm theoretical basis. Having computed the stress relaxation function, the complex modulus $G^{*}\left(\omega \right)$, storage modulus $G^{\prime }\left(\omega \right)$, and loss modulus $G^{\prime \prime }\left(\omega \right)$ may also be derived from the following Fourier transform:

(24) $$G^{*}\left(\omega \right)=G^{\prime }\left(\omega \right)+iG^{\prime \prime }\left(\omega \right)=i\omega \int_{0}^{+\infty }\exp\left(-i\omega t\right)G(t)dt\;$$

Whereas G(t) refers to the time domain, the dynamic mechanical moduli of Equation (24) refer to the frequency domain. The frequency-dependent storage and loss moduli are measured experimentally and are very important for validating model predictions.

In this study, as mentioned earlier, crosslinked polymer networks are also simulated under one-dimensional deformation, i.e., elongation and compression along a predefined direction. The stress of elastomeric materials under the aforementioned deformations is usually measured either as a function of the true strain, *ε*, or of the engineering strain, *e* (also known as Cauchy strain). The aforementioned strains are connected through the relation ε=ln(1+e)=ln(λ), where *λ* is the relative elongation, and for rather small elongations the approximate equality ε≈e holds. The stress-strain curves may be plotted by conducting simulations at different strains and recording the stresses. Thus, the elastic (Young’s) modulus *E* can be computed as the slope of the linear region of the stress-strain curves at low deformations.

In the analysis below, it is assumed that an elastomeric material of rectangular parallelepiped shape, with edges *L_x_*, *L_y_* and *L_z_*, is elongated along the *z*-axis which causes an increase in length by Δ*L* in this direction with simultaneous contraction by $\Delta L^{\left(x\right)}$, $\Delta L^{\left(y\right)}$ along the *x* and *y* axes, respectively. The infinitesimal strains are defined as:

(25) $$d\varepsilon_{x}=\frac{dx}{x},\;d\varepsilon_{y}=\frac{dy}{y},\;d\varepsilon_{z}=\frac{dz}{z}\;$$

In this case, the Poisson ratio is given by the next equations:

(26) $$v=-\frac{d\varepsilon_{y}}{d\varepsilon_{z}}=-\frac{d\varepsilon_{x}}{d\varepsilon_{z}}\;$$

Making use of Equation (25) and assuming constancy of *ν* over the integrating ranges, the following equations are derived:

(27) $$-v\int_{L_{z}}^{L_{z}+\Delta L}\frac{dz}{z}=\int_{L_{y}}^{L_{y}-\Delta L^{\left(y\right)}}\frac{dy}{y},\;-v\int_{L_{z}}^{L_{z}+\Delta L}\frac{dz}{z}=\int_{L_{x}}^{L_{x}-\Delta L^{\left(x\right)}}\frac{dx}{x}\;$$

which leads to:

(28) $${\left(1+\frac{\Delta L}{L_{z}}\right)}^{-v}=1-\frac{\Delta L^{\left(x\right)}}{L_{x}},\;{\left(1+\frac{\Delta L}{L_{z}}\right)}^{-v}=1-\frac{\Delta L^{\left(y\right)}}{L_{y}}\;$$

The relative elongation is defined as $\lambda =(L_{z}+\Delta L)/L_{z}$, whereas for an initially cubic box (before deformation) $L=L_{x}=L_{y}=L_{z}$ holds. After deformation of the box, the new lengths along the *x*-, *y*- and *z*-directions are denoted by $L_{x}^{\prime }$, $L_{y}^{\prime }$, and $L_{z}^{\prime }$, respectively. Therefore, the following set of relations is obtained:

(29) $$\begin{array}{l} L_{x}^{\prime }=\lambda^{-v}L_{x}\\ L_{y}^{\prime }=\lambda^{-\nu }L_{y}\\ L_{z}^{\prime }=\lambda L_{z} \end{array}$$

The volume of the deformed box is related to the volume of the (initial) cubic box by:

(30) $$V^{\prime }=\lambda^{1-2\nu }V\;$$

If one sets *ν* equal to 0.5, the elongation is volume-preserving as indicated by the last equation. An interesting review article about the Poisson ratio of modern materials can be found in reference [90]. In our simulations, following a change in the edge lengths of the box, the positions of all nodal points are transformed affinely according to the relation:

(31) $$\begin{array}{l} x^{\prime }=\lambda^{-v}x\\ y^{\prime }=\lambda^{-\nu }y\\ z^{\prime }=\lambda z \end{array}$$

This transformation takes place when all nodal points have been folded back to the primary simulation box [36]. In the context of rubber-like elasticity theory, the elastic modulus *E* may be computed by a least-squares fitting of stress-strain data to a straight line according to the model [65,91]:

(32) $$\sigma =G\left(\lambda^{2}-\lambda^{-1}\right)=\frac{E}{3}\left(\lambda^{2}-\lambda^{-1}\right)$$

(33) $$\sigma =\sigma_{zz}-\frac{\sigma_{xx}+\sigma_{yy}}{2}\;$$

where *σ* is computed as the difference between stress in the stretching direction and normal stress in the transverse directions. For uniaxial deformation experiments, the Mooney–Rivlin expression is employed as well [91]:

(34) $$f^{*}=2C_{1}+\frac{2C_{2}}{\lambda },f^{*}(\lambda^{-1})=\frac{\sigma }{\lambda^{2}-\lambda^{-1}}\;$$

This is a two-parameter model (*C*_1_, *C*_2_) with the Mooney stress being defined by the second of Equations (34). The Mooney–Rivlin expression is not appropriate for (uniaxial) compression experiments and does not give very good fits for a wide range of relative elongations in a uniaxial deformation experiment [68]. A more powerful model that is free of these drawbacks was introduced by Rubinstein and Panyukov and is known as the slip-tube model in the literature. The simple analytical approximation of this model, which is provided by Equation (35), allows one to separate the phantom and entanglement contributions to the Mooney stress f*(*λ*^−1^) [68]:

(35) $$\frac{f\;*(\lambda^{-1})-G_{c}}{G_{e}}=\frac{1}{0.74\lambda +0.61\lambda^{-1/2}-0.35}\;$$

The two moduli (*G*_c_, *G*_e_) comprise the only adjustable parameters in the context of this model.

## 3. Details about the Engine for Mesoscopic Simulations for Polymer Networks (EMSIPON) Code

**Scheme 1:**
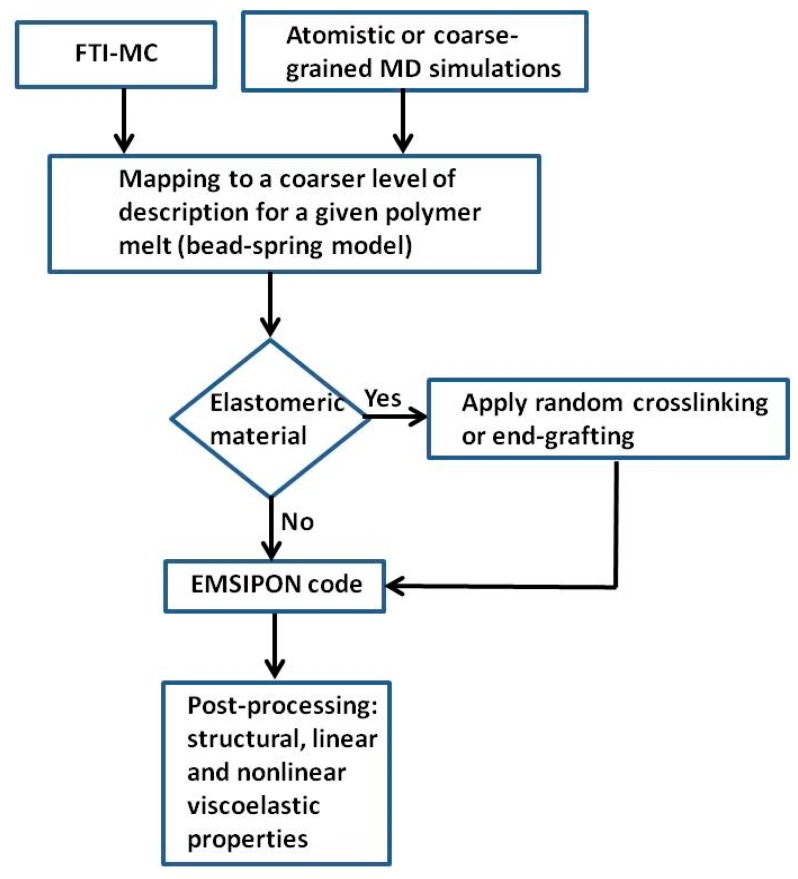
Flow diagram for the presented multiscale approach.

The methodology described above is implemented in a C++ code (EMSIPON) developed by the authors of this article in the *Computational Materials Science and Engineering Group* at the National Technical University of Athens. An overview of our multiscale approach is provided in the flow diagram of Scheme 1. EMSIPON has been written in object-oriented style with input/output configurations and trajectories being saved in the format adopted by the well-known LAMMPS (Large-Scale Atomic-Molecular Massively Parallel Simulator) [92]. Also, code has been developed for the computation of several properties which are recorded in a number of output files. More specifically, EMSIPON performs the following functions:

(a) Generation of a network configuration. Crosslinked polymer networks are generated in two different ways, i.e., random-crosslinking and end-grafting. Both are applied on equilibrated polymer melts, obtained here by FTi-MC simulations of dense systems of chains represented as freely jointed sequences of Kuhn segments using a Helfand Hamiltonian for non-bonded interactions [77,78]. The interconnection of EMSIPON with FTi-MC gives rise to many possibilities for bottom-up coarse graining of polymer systems. Also, crosslinking can be applied on equilibrated polymer melts created by atomistic or coarse-grained molecular dynamics and Monte Carlo simulations.

(b) Dynamical simulation of a network at rest. The motion of nodes in three-dimensional space is tracked with Brownian dynamics, while the slip spring hopping, creation and destruction mechanisms through which entanglement effects are introduced are implemented by kinetic Monte Carlo, as described in Section 2.3. A principal aim of the EMSIPON code is the computation of viscoelastic properties of polymer networks in the presence or absence of permanent crosslinks, through the computation of the stress relaxation function. Furthermore, a full analysis of structural properties can be performed in order to check whether unperturbed chain conformations are adopted by the strands.

(c) Dynamical simulation of mechanical deformations of crosslinked polymer networks. This additional option is offered to conduct uniaxial tension (compression and elongation) in silico experiments on elastomeric materials of a given Poisson ratio (normally, *ν* = 0.5). Besides, special attention is paid to the study of ordering at the level of strands, slip springs and longer segments (e.g., subchains connecting successive crosslinks), as a function of the imposed strain (see also Results section).

(d) Dynamical non-equilibrium simulation of polymer melts under shear flow [50]. Such simulations make use of Lees–Edwards periodic boundary conditions [93], over a broad range of Weissenberg numbers. This approach, which is complementary to equilibrium simulations, is employed for the computation of non-linear rheological properties (e.g., shear thinning at large shear rates, first and second normal stress differences, stress overshoot upon startup of shear flow, etc.).

Moreover, the aforementioned code can be extended to mesoscopic simulations, in a bottom-up fashion, of more complex polymer-based systems, such as associative polymer networks (formed by temporary bonds undergoing reversible association and disassociation), polymer-matrix nanocomposite materials, and polymer melts in contact with planar surfaces or solid inclusions. It should also be pointed out that our methodology, as presented in detail herein and in two other references [49,50], can only be implemented by EMSIPON; our kMC schemes and the field-based non-bonded interactions associated with an EOS are not available in existing simulation packages (e.g., LAMMPS) or other codes oriented for coarse-grained simulations of polymer systems, such as NAPLES (New Algorithm for Polymeric Liquids Entangled and Strained) which implements the well-known PCN (Primitive Chain Network) model [34,37,39]. Furthermore, our code incorporates algorithms described in other related articles; for instance the compensating potential introduced by Chappa et al. [43] to prevent perturbations in chain conformations caused by the slip-springs. The effectiveness of our methodology and the corresponding code is proven by comparison of the results with theory and existing experimental data. In addition, our methodology can be combined with the IBI method [4,5] and the force-matching (FM) method [94,95,96]. In Scheme 1, we mention that the starting point can be a coarse-grained model that may be derived from either a systematic coarse-graining method or a “top-down” approach. However, the disadvantage of the IBI and FM methods is that the configurational free energies are temperature dependent. In our approach, due to the use of an EOS for the computation of the field-based non-bonded interactions, we can simulate polymer melts and crosslinked polymer networks in a wide range of temperatures, densities and pressures, different to those of the equilibrated initial configuration that is used as input in EMSIPON, without resorting back to the atomistic or the more detailed coarse-grained model.

## 4. Simulations

A monodisperse *cis*-PI melt consisting of $n_{\mathrm{chains}}=$172 linear chains is initially simulated by a field-theory inspired Monte Carlo [77,78] method at the level of Kuhn segments (freely jointed chain model) with each chain comprised of 530 Kuhn segments. When an equilibrated configuration is obtained, we proceed with coarse-graining to the level of a few Kuhn segments per bead as detailed above. In the random crosslinking case, two polymer networks are created by dispersing *n*_c_ = 300 and *n*_c_ = 600 crosslinks in the simulation box and capturing pairs of neighboring chains at each crosslink. The percentage of crosslinks in the elastomeric material is defined as *p*_c_ = *n*_c_/*n*_bead_ × 100%, where *n*_bead_ is the total number of beads in the system under consideration. A visualization of the whole polymer network is given in Figure 1b. The friction coefficient can be calculated either from temperature-dependent expressions fitted to experimental data [49], or from analyses of the temperature-dependent chain self-diffusivity and Rouse time based on atomistic molecular dynamics simulations of unentangled melts [97]. Here, an experimental value of the friction factor per monomer at the simulation temperature (based on a set of Williams–Landel–Ferry parameters) is chosen ($\zeta_{0}$ = 1.15 × 10^−11^ kg/s) [49]. All Brownian dynamics simulations are conducted at constant temperature (*T* = 400 K) and volume with the integration time step (Δ*t*) being equal to 50 ps. This is the time step employed in the simulations of reference [50] and gives identical structural and dynamical properties compared with simulations with shorter time steps (i.e., 1 and 5 ps). In particular, the crosslinked polymer network in the undeformed state is simulated in a cubic box of edge length $L_{x}=L_{y}=L_{z}=30\;\mathrm{nm}$ and a cubic grid containing 5 × 5 × 5 = 125 equal-size cells is introduced for the calculation of non-bonded interactions. The characteristic size of the nodal points ($R_{j}$) is chosen as 3.06 nm; this is the edge length of the cube, centered at each nodal point, in which the amount of matter associated with the nodal point is assumed to be uniformly distributed. The Sanchez–Lacombe parameters $\rho^{*}$, $P^{*}$ and $T^{*}$ for crosslinked *cis*-PI networks are taken from reference [98] and are used here without any modification. In the uniaxial tension simulations of the polymer networks, the simulation box has the shape of a rectangular parallelepiped and the number of grid cells is maintained constant. All these virtual deformation experiments are conducted under constant volume and consequently the Poisson ratio (*ν*) is considered equal to 0.5. The crosslinked polymer networks under study are simulated in a broad range of relative elongations with respect to the initial undeformed box dimensions $\left\{\lambda_{\min}=0.70,\;\lambda_{\max}=2.10\right\}$. All the simulation parameters are gathered in Table 1. As far as the hopping scheme for slip springs is concerned, the parameter *ν*_diff_ (see also Equation (18)) is set to 10.0 s^−1^, a value falling in the range from 0.1 to 10.0 s^−1^, as suggested by Vogiatzis et al. in reference [49]. Concerning the entanglement effect, this is introduced by a predefined number of slip springs, i.e., 900 and 1200. Two different values are employed so as to check the effect of slip springs on the viscoelastic properties. In addition, some results from crosslinked polymer networks without slip springs are presented as well. Before closing this section, it should be mentioned that when different parameters are employed in the simulations, they will be provided in the discussion of the results in the next section.

## 5. Results

**Figure 4:**
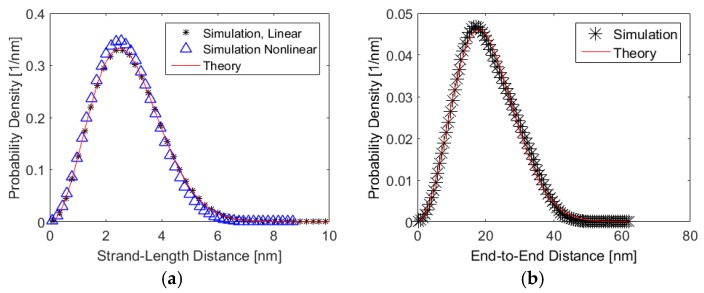
Strand length (**a**) and end-to-end distance (**b**) distributions for a crosslinked polymer network created by random crosslinking. More details are provided in the main text.

As already mentioned above, this paper concerns an extension of the methodology and the corresponding C++ code of references [48,49,50] to the simulation of elastomeric materials in the undeformed and deformed states. To this end, a number of crosslinked polymer networks have been studied with the simulation details being provided in Section 4 and Table 1. Most of the results presented here concern networks created by random crosslinking. Some results from end-grafted networks have been presented elsewhere [48]. The analysis begins with the computation of selected conformational properties. Here our principal aim is to check whether the unperturbed chain conformation of the initial configuration (as obtained from the FTi-MC simulation) is retained during the Brownian dynamics/kinetic Monte Carlo simulations. First, the strand length distribution and the end-to-end distance distribution of the originally linear chains that formed the network are presented and compared with the corresponding theoretical predictions given by the following equations, respectively:

(36) $$\begin{array}{c} P\left(r\right)={\left(\frac{3}{2\pi n_{\mathrm{Kuhn}/\mathrm{strand}}b^{2}}\right)}^{3/2}\exp\left(-\frac{3}{2}\frac{r^{2}}{n_{\mathrm{Kuhn}/\mathrm{strand}}b^{2}}\right)\\ P\left(r\right)=4\pi r{}^{2}P\left(r\right) \end{array}$$

(37) $$\begin{array}{c} P^{\mathrm{end}\text{-}\mathrm{to}\text{-}\mathrm{end}}\left(r\right)={\left(\frac{3}{2\pi n_{\mathrm{Kuhn}/\mathrm{strand}}n_{\mathrm{strands}/\mathrm{chain}}b^{2}}\right)}^{3/2}\exp\left(-\frac{3}{2}\frac{r^{2}}{n_{\mathrm{Kuhn}/\mathrm{strand}}n_{\mathrm{strands}/\mathrm{chain}}b^{2}}\right)\\ P^{\mathrm{end}\text{-}\mathrm{to}\text{-}\mathrm{end}}\left(r\right)=4\pi r{}^{2}P{}^{\mathrm{end}\text{-}\mathrm{to}\text{-}\mathrm{end}}\left(r\right) \end{array}$$

with *b* being the Kuhn length of the simulated polymer, $n_{\mathrm{Kuhn}/\mathrm{strand}}$ the number of Kuhn segments per strand, and $n_{\mathrm{strands}/\mathrm{chain}}$ the number of strands per polymer chain. In the context of this study, a single value for the latter parameter indicates that the polymer network is created from a monodisperse polymer melt. The effect of polydispersity on the rheological properties of polymer melts and rubbers is outside the scope of this article and shall comprise the topic of a future study. The presented distributions in Figure 4a,b concern a randomly crosslinked polymer network with *p*_c_ = 7.0% and *n*_slip-springs_ = 900 and it is seen that the agreement between simulation data and corresponding theoretical predictions, as described by Equations (36) and (37), is very good. In Figure 4a, another distribution (triangles in blue color) is also included, corresponding to the same system, but with the linear entropic springs replaced by finitely extensible non-linear ones whose analytical force law is provided by Equation (3). The distributions for linear and non-linear springs are expected to be quite similar for elastomeric materials in the undeformed state or at small deformations; indeed, they turn out to be very similar. Results from a slightly entangled crosslinked polymer network with *n*_c_ = 300 have been presented elsewhere [48]. In that case, the end-to-end distance distribution was even closer to the theoretical prediction of Equation (37) due to the rather low crosslink and slip spring densities. In general, the deviation from the theoretical curve of Equation (37) becomes smaller as the number of crosslinks and slip springs is reduced. It is noted that for highly entangled polymer networks, a compensating potential [43] can be implemented to cancel completely the effect of slip springs on the chain conformations of the polymer melt or network. Although the option of using a compensating potential is included in the EMSIPON code, it should be activated only when absolutely necessary, in view of the computational burden it entails; in most cases a large number of pairwise additive interactions has to be calculated [50].

**Figure 5:**
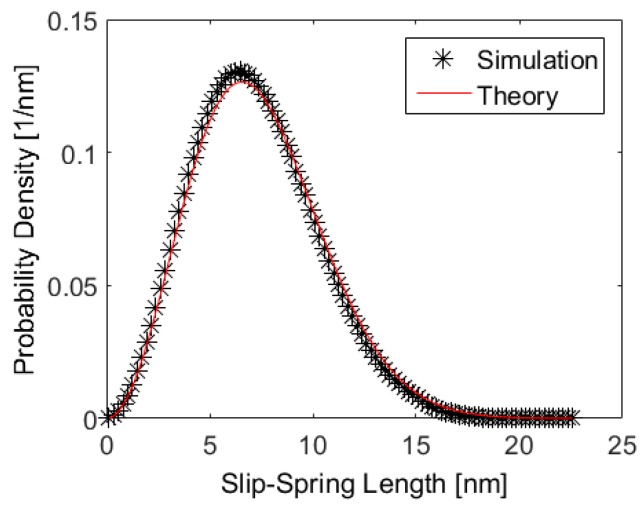
Slip spring length distribution of a crosslinked polymer network created by random crosslinking.

**Figure 6:**
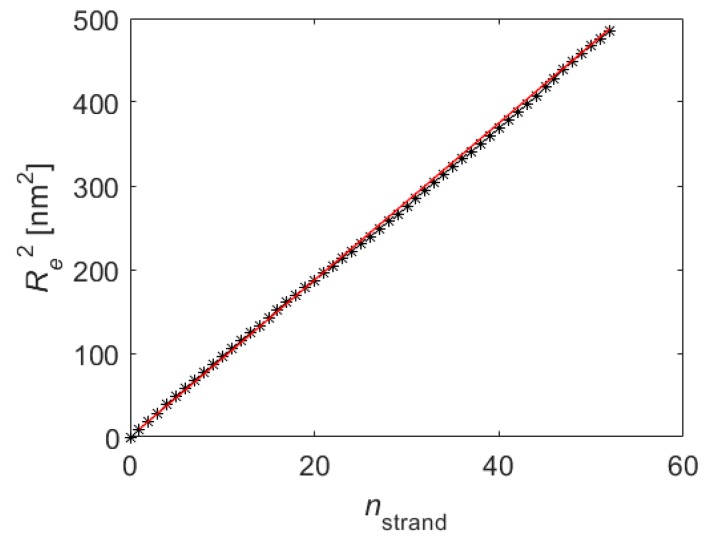
Real distances of nodal points along the chains (see also Equation (38)) as a function of the number of strands (*n*_strand_) for a crosslinked polymer network (this is the same crosslinked network of Figure 4 and Figure 5).

In addition to the aforementioned distributions, we have computed the slip spring length distribution, which is again compared with the corresponding theoretical prediction derived from Equation (36) with the term $n_{\mathrm{Kuhn}/\mathrm{strand}}b^{2}$ replaced with an effective mean squared end-to-end distance of slip springs (the target value for the latter is equal to the squared entanglement tube diameter of the simulated polymer). The distribution in question, depicted in Figure 5, is Gaussian, in very good agreement with theoretical expectations. The unperturbed conformation of chains is also probed by another property, defined as follows:

(38) $$R_{e,i}^{2}=\langle {\left|r_{j+i}-r_{j}\right|}^{2}\rangle ,\;j=1,\;2,\ldots ,n_{\mathrm{beads}/\mathrm{chain}},\;i=0,1,2,\ldots ,\;n_{\mathrm{beads}/\mathrm{chain}}-1\;$$

with the average being taken over all *j*. This measure concerns the squared distances, in three dimensions, between nodal points along polymer chains constituting the crosslinked polymer network. Its importance lies in the fact that it provides a full description of all possible distances among beads in the same chain. The $R_{e,i}^{2}$, is initially plotted as a function of the parameter *n*_strand_, which is the number of strands between two given beads on the same chain. In Figure 6, $R_{e,i}^{2}$, as obtained from the mesoscopic simulations of the considered network (black asterisks), is compared against the quantity $n_{\mathrm{strand}}n_{\mathrm{Kuhn}/\mathrm{strand}}b^{2}$, which is depicted with a red straight line; clearly, there is excellent agreement. It is pointed out that the accordance of all conformational properties examined above with the corresponding theoretical predictions is comparable to that observed in BD/kMC simulations of monodisperse polymer melts of *cis*-PI, presented in detail in reference [49].

If chain conformations in the network are unperturbed, the relative orientation of successive strands along a chain should be random. This is checked in the simulation by computing the average angle $\bar{\theta }=\cos^{-1}\left(\langle \cos\theta \rangle \right)$ between two successive strands or, equivalently, three successive nodal points along a polymer chain. The expected value is 90°, corresponding to a polymer network of completely random orientations. The $\bar{\theta }$ angles are provided in Table 2 for two polymer networks containing 300 and 600 permanent links and it is seen that there is very good agreement between the expected and simulation values.

An important question is whether the isothermal compressibility of the polymer networks is calculated correctly; capturing this property is one of the main reasons why non-bonded interactions are included in our BD/kMC simulations. As reported in Section 2.1, the excess Helmholtz energy density of the Sanchez–Lacombe EOS is employed in the non-bonded free energy. The expected value of the isothermal compressibility for this equation of state is:

(39) $$\kappa_{T}^{\mathrm{Sanchez}\text{-}\mathrm{Lacombe}}=-\frac{1}{V}{\left(\frac{\partial V}{\partial P}\right)}_{T,n_{\mathrm{chains}}}={\frac{1}{P^{*}\overset{}{\tilde{\rho }}\left(\frac{\partial \tilde{P}}{\partial \tilde{\rho }}\right)}}_{\tilde{T}}\;$$

From the simulation point of view, the property in question is computed by the following equation, stemming from an analysis of density fluctuations in the grand canonical ensemble at equilibrium [99,100]:

(40) $$\kappa_{T}=\frac{V_{\mathrm{cell}}\langle {\left(\delta n_{\mathrm{Kuhns}/\mathrm{cell}}\right)}^{2}\rangle }{k_{B}T{\langle n_{\mathrm{Kuhns}/\mathrm{cell}}\rangle }^{2}}\;$$

The averages 〈…〉 are taken over all cells of the grid which has been introduced in the simulation box for the computation of non-bonded interactions. More specifically, the averages in the numerator and denominator of Equation (40) are the variance and the mean value of the number of Kuhn segments in each cell, respectively. The temporal averages of isothermal compressibilities for all polymer networks, as obtained from the Brownian dynamics simulations, are provided in Table 2 and the agreement with the estimated value from the Sanchez–Lacombe equation (1.29 × 10^−4^ bar^−1^) is very good at the density and temperature of the simulated networks. The fact that we predict the isothermal compressibility is an important advantage of our coarse-grained model, since this is missing from some other models available in the literature.

**Figure 7:**
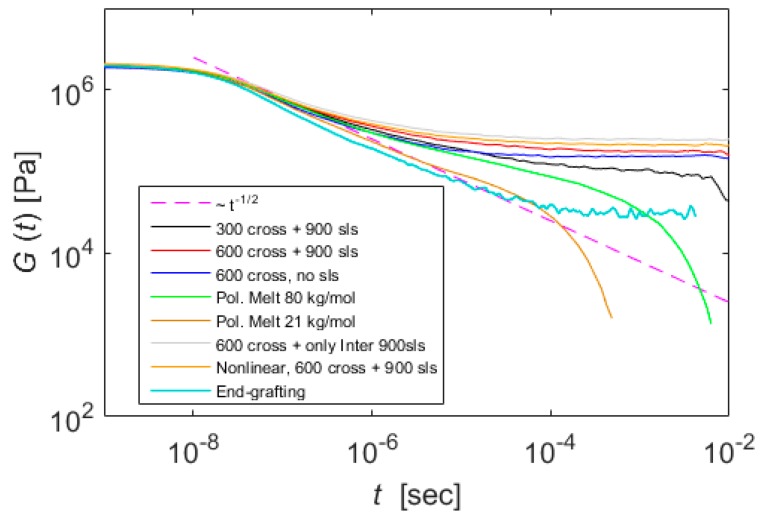
Stress relaxation functions of various polymer networks at different crosslink and slip spring densities obtained from random crosslinking. Two curves corresponding to *cis*-PI polymer melts consisting of monodisperse linear chains of different molar masses (i.e., 80 kg/mol and 21 kg/mol) are also included to indicate the different behavior between crosslinked and uncrosslinked polymer networks. In the case of these two curves, the stress relaxation functions were fitted to sums of Maxwell modes [49].

The stress relaxation function (see also Equation (23)) plays a central role in our analysis, since it is the starting point for the calculation of important linear rheological properties from the fluctuations of the off-diagonal elements of the stress tensor. As in the case of polymer melts [49,50], we computed this function for a number of crosslinked polymer networks in the undeformed state. In this study, all *G*(*t*)’s presented in Figure 7 were computed with the multiple tau-correlator algorithm [88]. Several curves are presented in Figure 7. Stress relaxation functions of two different polymer melts, as obtained in reference [49], are included for highlighting the different rheological behavior between crosslinked polymer networks and uncrosslinked melts at longer times. The molar masses of the chains comprising the two monodisperse melts are written in the legend of the figure and also the Rouse model scaling (~*t*^−0.5^) is included. At longer times (“terminal” region), the stress relaxation function drops exponentially for the polymer melts, in contrast to the elastomeric materials, which approach a constant value. This constant is the shear modulus *G* of the elastomer. In Figure 7, results from polymer networks with different architectures, crosslink and slip spring densities are presented. The trend is an increase in the stress relaxation function upon increasing the crosslink and slip spring densities. For a given number of slip springs, the exclusion of intramolecular ones (connecting beads on the same polymer chain) shifts the shear modulus upwards (compare blue and black curves). Furthermore, a crosslinked polymer network without any entanglements was studied so as to compute the shear modulus due to crosslinks only (*G* = *G*_c_).

In addition to the simulations of undeformed elastomeric materials and the computation of mechanical properties from fluctuations of the diagonal elements of the stress tensor, virtual one-dimensional experiments were conducted in which the strain (or the relative elongation) was imposed and the true stress was recorded (see also Equation (33)). In these simulations, only the diagonal elements of the stress tensor are taken into account. The elongation and compression experiments are able to reveal useful information from the microscopic point of view as a function of the imposed strain. In general, the rubbery materials are assumed to have a Poisson ratio equal to 0.5 and, therefore, the connection of shear (*G*) and elastic modulus (*E*) is expressed as E=2G(1+v)=3G (it is valid for isotropic materials). In the present study, this assumption is corroborated by computing the Poisson ratio from the shear modulus and the bulk modulus (*B*) which is the inverse of the isothermal compressibility, $\kappa_{T}$, in the undeformed system:

(41) $$v=\frac{3B-2G}{2\left(3B+G\right)}=\frac{3-2\kappa_{T}G}{2\left(3+\kappa_{T}G\right)},\;\kappa_{T}=\frac{1}{B}\;$$

**Figure 8:**
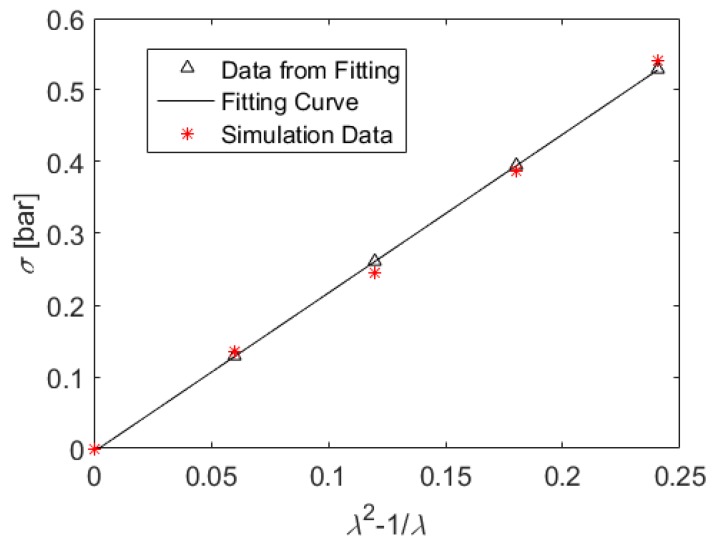
Elongation experiment at small deformations with respect to the undeformed state. Stress-strain simulation data were fitted to Equation (32).

The Poisson ratio comes out as 0.499 and consequently the value *ν* = 0.5 used in the virtual deformation experiments is fully justified. In order to compute the shear modulus, we have conducted elongation experiments at rather small relative elongations (1.00≤λ≤1.08) and fitted the simulation data to Equation (32). Such a plot is presented in Figure 8, in which the real stress (as defined in Equation (33)) is recorded as a function of the quantity *λ*^2^ − 1/*λ*. This diagram was produced by elongation experiments along the *x*, *y* and *z* directions and averaging the corresponding true stresses at a given *λ*. Simulations along only one direction may give real stresses that are not very close to zero at *λ* = 1, however, the estimated shear modulus computed from the slope of the fitted straight line is practically the same in all directions. It is reminded that all stress relaxation functions were calculated by averaging over all off diagonal elements of the stress tensor (*τ_xy_*, *τ_xz_*, *τ_yz_*). It is also mentioned that all relative elongations (either larger or smaller than unity) are computed with respect to the initial undeformed state.

In addition to elongation experiments at rather small strains, we have conducted virtual one-dimensional deformation experiments over a broad range of relative elongations (for more details see also Section 4). In reference [68], two sets of fitting parameters (moduli) are provided concerning natural rubber ($G_{c}≃1.77\times 10^{5}\;\mathrm{Pa}$, $G_{e}≃1.77\times 10^{5}\;\mathrm{Pa}$ and $G_{c}≃8.9\times 10^{4}\;\mathrm{Pa}$, $G_{e}≃8.1\times 10^{4}\;\mathrm{Pa}$). These two sets of values correspond to the shear moduli $G≃3.54\times 10^{5}\;\mathrm{Pa}$ and $G≃1.70\times 10^{5}\;\mathrm{Pa}$, respectively. All crosslinked and entangled polymer networks under consideration with *p*_c_ = 7.0% exhibit shear moduli falling in the range $(1.5-2.5)\times 10^{5}\;\mathrm{Pa}$.

A central role in our analysis of ordering is played by the Saupe ordering tensor [101], defined by the following equation:

(42) $$Q=\frac{1}{N}\sum_{i=1}^{N}\left\{\frac{3}{2}{\hat{u}}_{i}{\hat{u}}_{i}-\frac{1}{2}1\right\}\;$$

The sum of the *N* elements can be over all strands, slip springs or longer chain segments (e.g., subchains between successive crosslinks), with ${\hat{u}}_{i}$ being a unit vector along the *i*-th segment. Moreover, the unitary tensor of second order is symbolized by 1. The (dyadic) tensor Q, which is widely employed for the study of liquid-crystalline phases [102], is obtained as an instantaneous value for each configuration. As defined in Equation (42), it is a symmetric tensor and therefore it will have three real eigenvalues. Furthermore, it is easily proved that it is traceless and, thus, the sum of its three eigenvalues equals zero. Segments of polymer melts and deformed crosslinked polymer networks at small deformations behave as isotropic phases; in this case, all the three real eigenvalues must be close to zero. Upon imposition of strong uniaxial tension, the segments are oriented in the direction of elongation, in a manner similar to that in which rod-like molecules in a nematic phase are oriented along the director. The largest eigenvalue of **Q** is the order parameter *S* [101,102]:

(43) $$S=\max\left\{\alpha_{i}\right\},\;i=1,2,3\;$$

**Figure 9:**
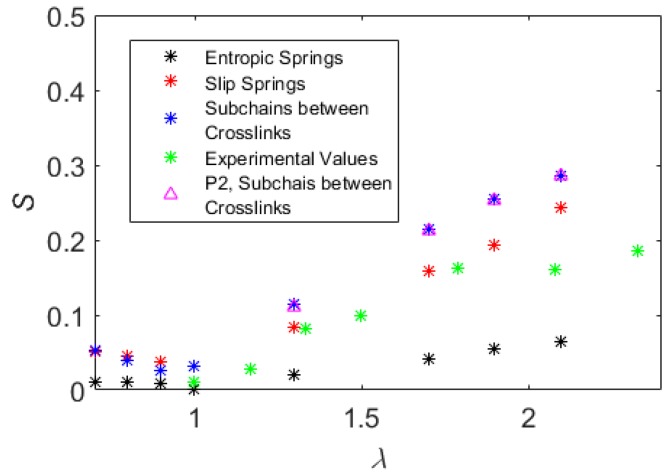
Largest eigenvalue of **Q** (parameter *S*) of a crosslinked polymer network containing 600 crosslinks and 900 slip springs as a function of the relative elongation *λ* (compression: *λ* < 1, elongation: *λ* > 1) at three different length scales, i.e., entropic springs (strands), slip springs, and subchains connecting successive crosslinks along the polymer chains. Order parameters, as defined from Equation (44), are also included for the simulated polymer network. Experimental values based on Fourier transform infrared spectroscopy (FTIR) are from [103,104].

At this point, it should be noted that this kind of ordering analysis may be also applied for polymer systems with solid inclusions. For instance, one can estimate to what extent a solid surface (e.g., graphene planes) causes orientational ordering in a polymer melt which is in contact with it. The analogy with liquid-crystals is the presence of defects or solid surfaces in a liquid-crystalline phase. In Figure 9, the order parameter *S* is plotted as a function of the relative elongation (*λ*) for chain segments at three different length scales, i.e., entropic springs, slip springs, and subchains connecting successive crosslinks along the polymer chains. The considered segments of the crosslinked polymer networks are expected not to be oriented along any preferential direction in the undeformed state, or at rather small deformations. This is clearly seen in Figure 9. As the polymer network is compressed or elongated along a predefined direction (*z*-axis), the order parameter increases. It is observed that the ordering is more pronounced for segments with bigger lengths as the imposed deformation increases. However, this enhancement is less pronounced in the case of virtual compression experiments (*λ* < 1). The reader is reminded that a one-dimensional compression experiment is equivalent to a two-dimensional elongation experiment along the other two orthogonal directions. In addition to the simulation data provided in Figure 9, some experimental measurements are included, as obtained from Fourier transform infrared spectroscopy (FTIR), quantified in terms of the following orientation function which is the Legendre polynomial of second order [103,104]:

(44) $$\langle P_{2}\left(\cos\theta \right)\rangle =\frac{1}{2}\left(3\langle \cos^{2}\theta \rangle -1\right)\;$$

where *θ* is the angle formed between a chain segment and the stretching direction. This order parameter is recorded as a function of the relative elongation and depicted with green asterisks; these values concern a highly crosslinked *cis*-PI polymer network with molar mass between crosslinks (*M*_c_) equal to 4500 g/mol and chain segments corresponding to roughly two Kuhn segments. It is pointed out that the smallest length scale in the context of our mesoscopic model is an entropic spring which corresponds to 10 Kuhn segments. To underline that the order parameter defined by Equation (44) and the largest eigenvalue of **Q** (Equation (43)) give very similar results for one-dimensional elongation experiments, we have plotted them simultaneously for segments connecting successive crosslinks (points in magenta and blue colors). An ordering analysis based on the Saupe ordering tensor becomes more informative when more complex deformation experiments are conducted (e.g., compression experiments, etc.). In these cases, more parameters are needed for a detailed description of the segmental ordering and the three real eigenvalues of **Q** comprise an excellent tool for such an analysis.

## 6. Discussion and Conclusions

A new methodology for mesoscopic simulations of crosslinked polymer networks and the corresponding C++ code have been presented in this article as an extension of our previous work concerning polymer melts. A polymer network is envisioned as a graph containing three kinds of beads: end-points, internal nodal points, and crosslinks. The latter are treated as permanent links between two different polymer chains, while the entanglement effect is introduced by a number of slip springs. The system thermodynamics and dynamics is derived from a Helmholtz free energy that incorporates local density-dependent non-bonded contributions calculated from an equation of state, as well as entropy spring contributions associated with all strands and slip springs between beads. The systematic force on each bead is derived as a gradient of this free energy function. More complex systems (e.g., containing nanoparticles or interfaces) can be described by adding more terms to the Helmholtz energy. The motion of beads in space is simulated with Brownian dynamics, whereas the hops of slip springs are tracked with a kinetic Monte Carlo scheme. Moreover, there are creation and destruction processes for slip springs at chain ends, which obey the condition of microscopic reversibility. The viscoelastic behavior of polymer networks is studied by computing the stress tensor as a function of time and autocorrelating it to accumulate the shear stress relaxation function. Furthermore, a procedure for simulating mechanical deformations has been tested in uniaxial tension computer experiments of crosslinked polymer networks. EMSIPON allows for studying various modes of crosslinking and their effects on properties and it is interconnected with the FTi-MC code developed for equilibrating coarse-grained models of dense polymer systems wherein linear chains are represented as sequences of Kuhn segments. The two codes together implement a systematic multiscale modeling approach for studying polymer networks at the mesoscale. Also, non-equilibrium Brownian dynamics/kMC simulations of polymer melts under shear flow may be conducted by employing Lees–Edwards periodic boundary conditions over a broad range of Weissenberg numbers. One of the main advantages of our methodology is that the number of adjustable parameters of our model is kept to an absolute minimum. Moreover, realistic time scales are simulated without introducing a posteriori validation against existing experimental findings of linear and non-linear rheology. It is noted that EMSIPON has been parallelized via MPI (message passing interface), but due to the scheme adopted for the non-bonded interactions, productive simulations can effectively run up to a few tens of cores. Massive parallelization will be achieved when a dissipative particle dynamics [105,106,107,108] integrator will be added to EMSIPON. This extension is under development and the field-based non-bonded interactions shall be replaced by pairwise non-bonded potentials. The EMSIPON code can be made available by the authors to interested readers upon request.

## Figures and Tables

**Table 1:** Parameters used in Brownian dynamics/kinetic Monte Carlo (BD/kMC) simulations of crosslinked polymer networks.

$n_{\mathrm{chains}}$	172
$n_{\mathrm{beads}/\mathrm{chain}}$	53
$n_{\mathrm{strands}/\mathrm{chain}}=n_{\mathrm{beads}/\mathrm{chain}}-1$	52
$n_{c}$	300 (*p_c_* = 3.4%), 600 (*p*_c_ = 7.0%)
$n_{\mathrm{slip}\text{-}\mathrm{springs}}$	900, 1200
*B*	0.958 nm
$R_{j}$	3.06 nm
$v_{\mathrm{diff}}$	10.0 s^−1^
$\zeta_{0}$	$1.15\times 10^{-11}\;\mathrm{kg}/s$
T	400 K
$\left\{L_{x}=L_{y}=L_{z}\right\}$	30 nm
Δt	50 ps
v	0.5
$T^{*}$	649.4 K
$P^{*}$	$2.92\times 10^{8}\;\mathrm{Pa}$
$\rho^{*}$	955 kg/m^3^

**Table 2:** Isothermal compressibilities and *θ* angles for polymer networks simulated in the context of this study.

	$\kappa_{T}$, 10^−4^ bar^−1^	$\theta =\cos^{-1}\left(\langle \cos\theta \rangle \right)$, deg
*p*_c_ = 7.0%, *n*_slip-springs_ = 900	1.27±0.01	89.78±0.02
*p*_c_ = 3.5%, *n*_slip-springs_ = 1200	1.28±0.01	89.87±0.01
